# SeqFeatR for the Discovery of Feature-Sequence Associations

**DOI:** 10.1371/journal.pone.0146409

**Published:** 2016-01-05

**Authors:** Bettina Budeus, Jörg Timm, Daniel Hoffmann

**Affiliations:** 1 Research Group Bioinformatics, Faculty of Biology, University of Duisburg-Essen, Essen, NRW, Germany; 2 Institute for Virology, University Hospital Düsseldorf, Düsseldorf, NRW, Germany; Georgia Institute of Technology, UNITED STATES

## Abstract

Specific selection pressures often lead to specifically mutated genomes. The open source software SeqFeatR has been developed to identify associations between mutation patterns in biological sequences and specific selection pressures (“features”). For instance, SeqFeatR has been used to discover in viral protein sequences new T cell epitopes for hosts of given HLA types. SeqFeatR supports frequentist and Bayesian methods for the discovery of statistical sequence-feature associations. Moreover, it offers novel ways to visualize results of the statistical analyses and to relate them to further properties. In this article we demonstrate various functions of SeqFeatR with real data. The most frequently used set of functions is also provided by a web server. SeqFeatR is implemented as R package and freely available from the R archive CRAN (http://cran.r-project.org/web/packages/SeqFeatR/index.html). The package includes a tutorial vignette. The software is distributed under the GNU General Public License (version 3 or later). The web server URL is https://seqfeatr.zmb.uni-due.de.

## Introduction

There is a widening gap between the surge of information rich sequence data, and the human resources available for analysis. This is a problem that severely hampers progress in biomedicine and other life sciences [1, 2]. Ideally, experimental or clinical researchers who are most familiar with and interested in their data should be enabled to analyze their data by themselves. While software for statistics and graphics, such as R [3] (http://www.R-project.org/), are freely available and well-suited for such analyses, the steep slope of the learning curve is often discouraging experimental and clinical researchers, who are fully occupied with managing experiments or clinical duties. A general and relevant field where this disparity has been expressed to the authors by clinical researchers, especially immunologists and virologists, is the association of features of clinical interest with sequences. A concrete example is the association of patients’ HLA (Human Leukocyte Antigen) types with substitutions in a viral protein sequenced from these patients, as a way of identifying T-cell epitopes and immune escape mutations [4]. There are powerful computational tools for the identification of associations between sequences and features, for instance in the domain of genome wide association studies [5], or next-generation sequencing exome or genome comparisons [6, 7], but these tools are optimized for specific application scenarios and not for ease of use in experimental or clinical laboratory settings.

We have developed the R-package SeqFeatR to allow experimental and clinical researchers easier access to the statistical and graphical capabilities of R for feature-sequence association studies. R was chosen since it is a powerful, free, open source suite that is available for all commonly used computing platforms.

SeqFeatR has been successfully introduced in several virological labs, and sequence-feature associations identified with SeqFeatR have been experimentally confirmed, as in the case of novel CD8^+^ T-cell epitopes in HCV [8], or compensatory substitutions outside such epitopes [9].

These published examples have used the feature “HLA type” and amino acid sequences. However, SeqFeatR is completely agnostic about the type of feature used, as long as it can be labeled unequivocally, and it also processes nucleotide sequences. Both will be demonstrated in section “Examples beyond HLA-sequence association”.

## Core functionality of SeqFeatR

Given a set of related nucleotide or amino acid sequences, such as variants of a gene from several patients with certain phenotypes, SeqFeatR discovers in those sequences positions that are statistically associated with a “feature”, for instance with one of the patient phenotypes. An example of a feature of great clinical importance is the HLA type of a patient. In a patient infected with highly variable virus, such as HIV, HCV, or HBV, the HLA system of that patient selects viral variants with immune escape mutations. Thus we can expect that mutations in viral genome sequences are associated with the patient feature “HLA type”. SeqFeatR detects such associations, in other words: it finds among all alignment positions those that have a statistically significant association with a given feature. Analogous to the association of single alignment positions vs. features, SeqFeatR allows for the screening of associations of position pairs or tuples with sequence features, though at higher computational cost.

Technically, SeqFeatR reads FASTA formatted multiple sequence alignments, with each sequence labeled in its header line with the name of the feature, for instance the HLA type of the patient from whom the respective viral sequence has been extracted. The alignment should contain sequences that are positive for the feature of interest, and sequences that are negative. SeqFeatR steps through all alignment columns and applies frequentist or Bayesian methods to detect associations with the feature.

SeqFeatR itself does not implement alignment functionality, since there are many excellent programs for multiple sequence alignments that can be used to turn sequence sets into multiple sequence alignments, for instance MAFFT [10], T-Coffee [11], or Clustal omega [12].

### Frequentist approach

In the frequentist approach used in SeqFeatR, Fisher’s exact tests [13] are applied to contingency tables for all letters of the relevant alphabet (amino acid or nucleic acids) at all alignment positions vs. sequence features. This most frequently requested type of analysis is fast and also provided by the SeqFeatR web server. Logarithmically scaled *p* values are plotted along the alignment with single position resolution (Manhattan plots), or averaged over epitope sized windows. These association analyses are potentially affected by high numbers of false positives due to multiple testing. Therefore, SeqFeatR offers methods for multiple testing corrections, from the very conservative Bonferroni correction to the more relaxed control of False Discovery Rates (FDRs) [14].

**Fig 1:**
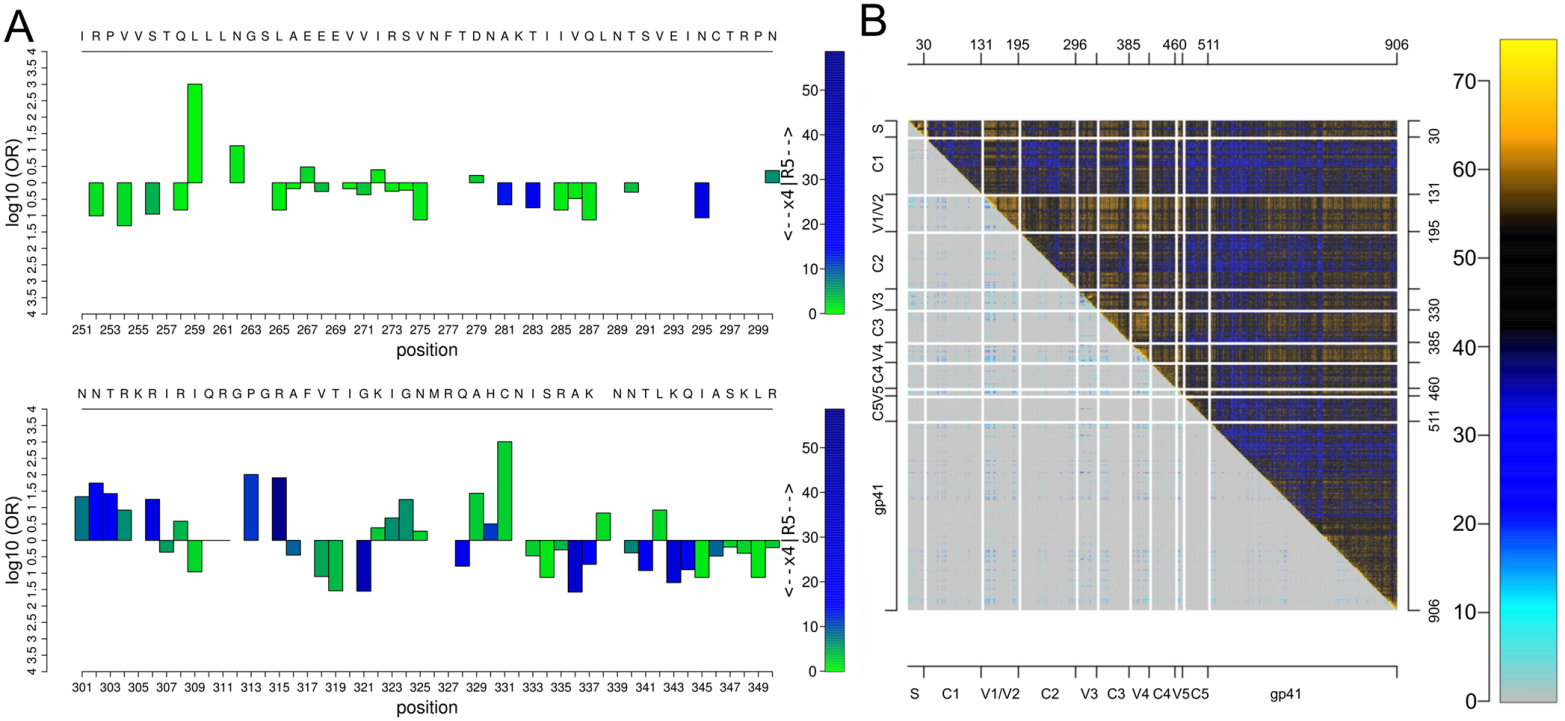
Odds-ratio plot and Tartan plot for visualization of statistical associations. **A** Odds-ratio plot, based on an alignment of region of HIV-1 gp120 around the V3 loop (C296-C331). Here, the feature is the predicted co-receptor tropism of HIV-1 [17] (R5 vs. X4 tropic). Bar heights and colors indicate logarithms of odds ratios and negative logarithms of *p* values, respectively. A reference sequence and sequence positions can be added in the top and bottom rows for orientation. **B** Tartan plot for the synopsis of two alignment pair association measures, here: −log *p* from association test between alignment position pairs (upper right triangle) vs. Direct Information between these pairs (lower left triangle). Association strengths are color coded (color legend on the right). For orientation, axes can be annotated and sequence substructures can be indicated by lines.

Beyond Manhattan plots, SeqFeatR provides some novel visualization tools for advanced exploratory analyses, for instance an odds-ratio plot that simultaneously shows, along a sequence, odds-ratios and *p* values as two aspects of association strength (Fig 1A). Another new visualization tool is the “Tartan plot” for a synopsis of two arbitrary scalar measures of sequence position pair association, e.g. (in Fig 1B) − log *p* from statistical association testing of amino acids at each pair (*i*, *j*) of alignment positions vs. the Direct Information between *i*, *j* [15, 16]. The synoptic plotting quickly reveals structure in such data, such as in Fig 1B the strong association of V1/V2 loops of HIV-1 gp120 protein with the other variable loops and parts of gp41, both in terms of *p* values from amino acid pair-association tests, and the more refined Direct Information.

### Bayesian approaches

While the frequentist approach works well in many cases, it has also drawn criticism, for instance because *p* values are often abused or misinterpreted [18]. Another problem with the frequentist approach occurs in situations where the same test is applied to multiple hypotheses, such as testing for associations with phenotype features for all positions in a multiple sequence alignment. As mentioned before, it is customary to “correct” the *p* values, e.g. by the very conservative Bonferroni correction or other more liberal alternatives, to avoid an increasing number of false positive tests results. As a more consistent alternative to deal with these problems, SeqFeatR offers also Bayesian inference methods [19], namely Bayes factors (BFs) and hierarchical models (though these are posing other problems, such as the necessity to specify priors). In the following we describe the implemented BF approach. For the hierarchical models we only mention that the SeqFeatR R-package has an interface to the Gibbs sampling engine JAGS [20]; a detailed account of hierarchical models for sequence feature association analyses will be given in a separate publication.

The BF for two hypotheses *H*_0_ and *H*_1_, given sequence and feature data *D*, is the ratio of posterior odds and the corresponding prior odds: *BF* = (*p*(*H*_1_|*D*)/*p*(*H*_0_|*D*))/(*π*_1_/*π*_0_). In other words, the BF equals the posterior odds ratio if the prior probabilities *π*_0_, *π*_1_ are equal and thus the prior odds ratio is 1. In our case *H*_1_ is the hypothesis that a feature is associated with an amino acid or nucleotide at an alignment position, and *H*_0_ is the hypothesis that there is no such association. The higher the BF, the more likely *H*_1_ (association) and the less likely *H*_0_ (no association). If the prior probability of association *π*_1_ is known, the ratio of posterior probabilities of association over non-association can be computed as *BF* ⋅ *π*_1_/(1 − *π*_1_).

Here we use a BF for the hypothesis *H*_1_ that feature and amino acid at an alignment position are *close* to independence vs. *H*_0_ that they are independent. A model *H*_1_ close to independence will often be more relevant than a “uniform model” that, for instance, assumes a uniform distribution of contingency table cell probabilities. Albert *et al*. have derived a BF expression for the ratio of a close-to-independence model over an independent model based on Dirichlet distributed elements of contingency tables [21, 22]:

$$\begin{array}{c} BF_{K}\left(\left\{y_{rc}\right\}\right)=\frac{\int \frac{\text{Dir}(\left\{K\eta_{r}\eta_{c}+y_{rc}\right\})}{\text{Dir}(\left\{K\eta_{r}\eta_{c}\right\})}d\left\{\eta_{r}\right\}d\left\{\eta_{c}\right\}}{\text{Dir}\left(\left\{y_{r}+1\right\}\right)\text{Dir}\left(\left\{y_{c}+1\right\}\right)}, \end{array}$$ (1)

where *y*_*rc*_ are the observed contingency table counts with row index *r* and column index *c*; $\text{Dir}\left(\left\{\alpha_{i}\right\}\right)=1/B\left(\left\{\alpha_{i}\right\}\right)\prod_{i}p_{i}^{\alpha_{i}-1}$ is the Dirichlet distribution of probabilities *p*_*i*_ (here: probabilities of contingency table elements) with normalizing multinomial Beta function *B* and concentration parameters *α*_*i*_; *y*_*r*_, *y*_*c*_ are the row and column sums of the observed contingency table; *K* is a precision hyperparameter; *η*_*r*_, *η*_*c*_ are hyperparameters corresponding to probabilities of row *r* and column *c* of tables with row-column independence. Curly brackets indicate that we have sets of two or more parameters. For instance, in the case of a 2 × 2 contingency table (amino acid present or absent at an alignment position versus feature present or absent), the Dirichlet distributions in the integrand depend on four parameters (two columns, two rows) and the integration therefore runs over four parameters. The prior belief in the independence is expressed by *K*: the higher this hyperparameter, the more dominant the independence structure imposed by *η*_*r*_ *η*_*c*_ will be in comparison to the observed counts *y*_*rc*_ in the numerator of Eq (1), and for *K* → ∞ complete independence is achieved. The BF is computed numerically as an average by importance sampling of Eq (1) using *η*_*r*_, *η*_*c*_ values that are randomly drawn from a Dirichlet distribution with concentration parameters evaluated from the entries *y*_*rc*_ of the observed contingency table. The procedure is detailed in Ref. [23]. *BF*_*K*_({*y*_*rc*_}) is reported by SeqFeatR.

While SeqFeatR allows for setting an explicit *K* value, it may not be easy to specify an appropriate value of *K* that is applicable to all alignment positions. In such cases, a new empirical Bayes variant of this BF is convenient. In this variant, an individual value of *K* is estimated from each contingency table itself. To derive this value, we first acknowledge that the sum *S* of absolute values of differences between the actually observed counts in the contingency table and the counts expected under independence is a measure of how confident we are that columns and rows are *dependent*:

$$\begin{array}{c} S=\sum_{rc}|y_{rc}-\frac{\sum_{k}y_{rk}\sum_{k}y_{kc}}{N}|, \end{array}$$ (2)

with total table count *N* = ∑_*rc*_ *y*_*rc*_. Clearly, for perfectly independent rows and columns, the value of *S* reaches its minimum of zero. The maximum of *S* = *N* is attained for strong dependence of rows and columns, for instance for a 2 × 2 table with *y*_11_ = *y*_22_ = *N*/2 = *n* and *y*_12_ = *y*_21_ = 0. To recast *S* into a measure of prior belief in *independence* we use as precision hyperparameter in Eq (1) instead of *K* the difference *K*_*D*_:

$$\begin{array}{c} K_{D}=N-S. \end{array}$$ (3)

A simple interpretation of *K*_*D*_ is that if all *N* counts in the contingency table support independence, we have *S* = 0 and therefore *K*_*D*_ = *N* (maximum prior belief in independence), while if all counts support association, we have *S* = *N* and therefore *K*_*D*_ = 0 (minimum prior belief in independence). SeqFeatR also offers the option of using *K*_*D*_.

**Fig 2:**
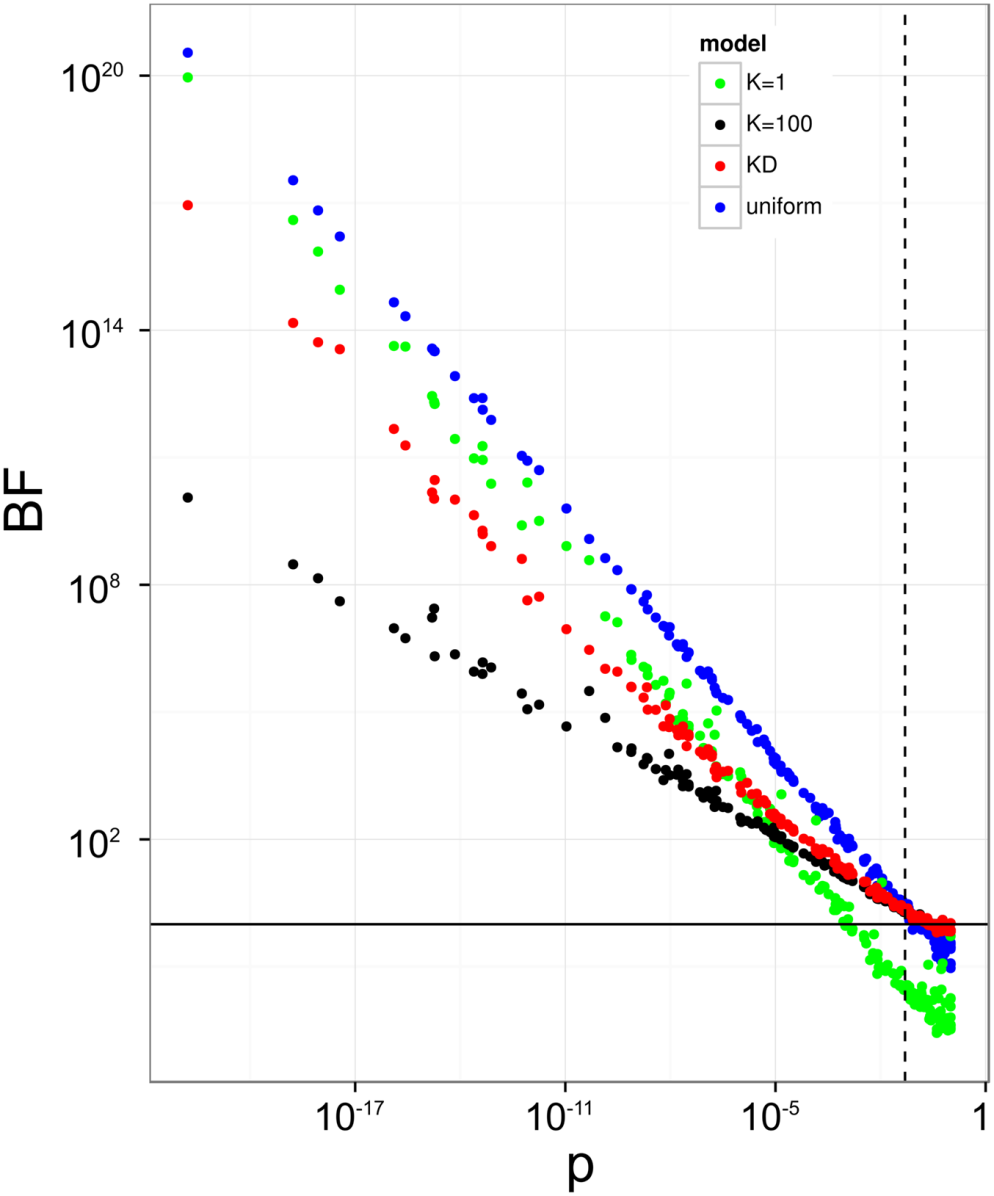
Comparison of statistical indicators of association. 200 random contingency tables with total count *N* = 100, a typical order of magnitude for analyses of sequence-feature association in practice, are analyzed by Fisher’s exact test, yielding *p* values for the rejection of independence (horizontal axis, not corrected for multiple testing), and by four different BF models, namely *K* = 1, *K* = 100, *K*_*D*_, and uniform model, with corresponding BFs on vertical axis. Solid horizontal black line at *BF* = 1 and dashed vertical line at *p* = 0.05 for orientation.

Fig 2 shows that for contingency tables for which independence cannot be rejected as indicated by *p* ≈ 1 from Fisher’s exact test (lower right corner), *K*_*D*_ and *K*_100_ yield approximately the same *BF* ≈ 1, i.e. association and independence are given approximately equal weights. In this corner, the uniform model and even more so the model with low confidence in independence (*K* = 1) have BFs much closer to zero, both favoring independence over dependence. At the other end of the *p* value range, on the left side of the plot, the low *p* values lead to rejection of independence, and concordant with this, high BFs that favor association over independence. Here, the increase of BFs in the *K*_*D*_ model follows those of lower *K* models. Effectively, the *K*_*D*_ model suppresses noise by collapsing weak-association cases to *BF* ≈ 1 (similar to high *K* models), while it readily supports stronger associations (similar to low *K* or uniform models).

### Comparison of frequentist and Bayesian approaches for discovery of HLA escape substitutions

**Fig 3:**
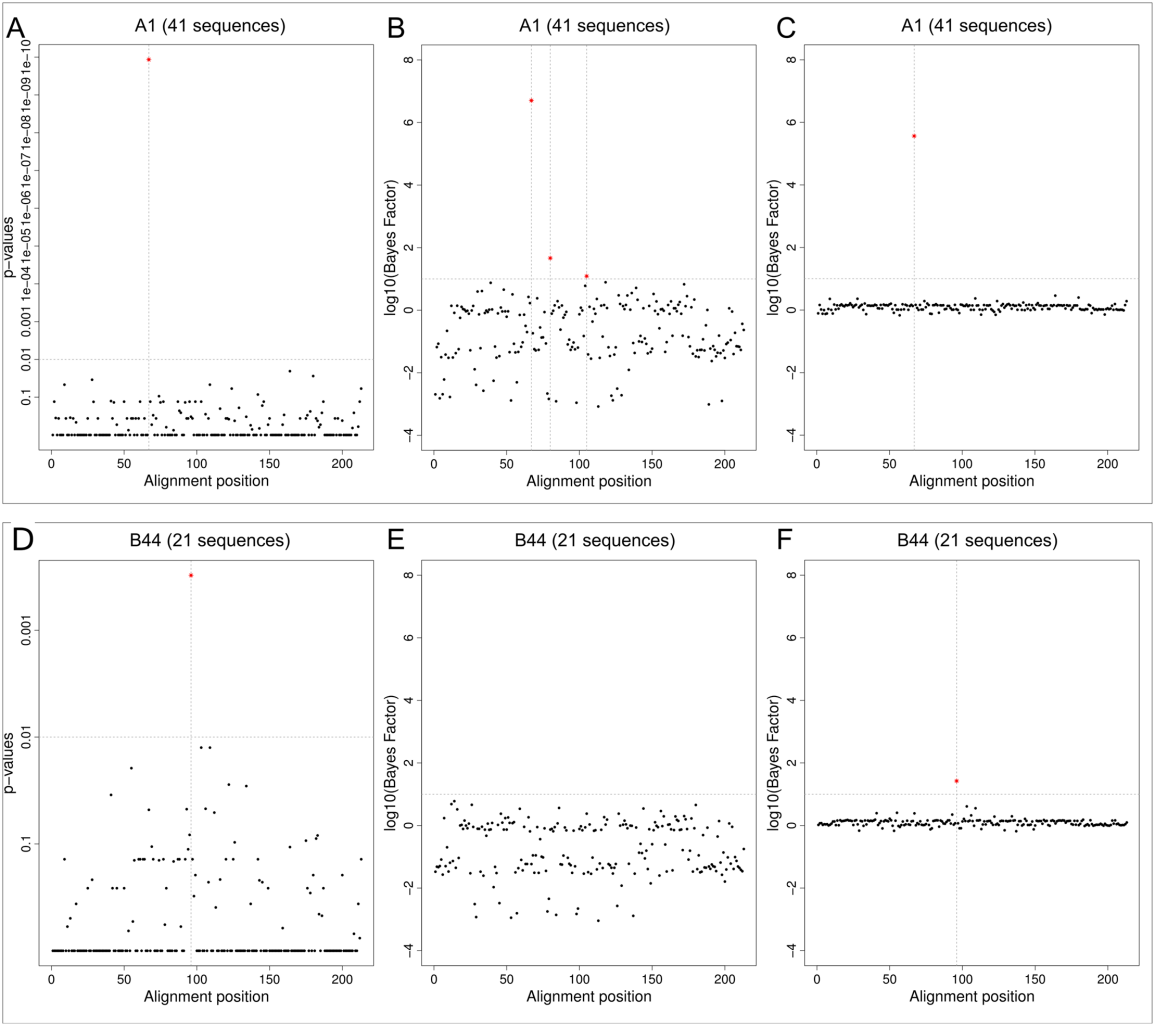
Comparison of frequentist approach and Bayes factors (BF). Discovery of association of alignment positions of HBV core proteins with patient HLA types, here: A*01 (top row) and B*44 (bottom row). Sequence numbers in panel titles are feature-carrying fractions of the total of 148 sequences included in the alignment. Association of sequences with feature HLA were analyzed by Fisher’s exact test (panels A, D), BF with *K* = 1 (panels B, E), and BF with *K*_*D*_ (panels C, F). Alignment positions with association above certain thresholds (horizontal dashed lines) are marked by red stars and vertical dashed lines, namely *p* < 0.01 (A, D), or *BF* > 10 (B, C, E, F). The *p* values and BFs shown are the best for each alignment position (lowest *p* values, highest *BF*s).

Recently, we have reported the discovery and experimental confirmation of several HLA escape substitutions in Hepatitis B Virus (HBV) from chronically infected patients [24] (sequences available from GenBank, accession numbers KP856971-KP857118). In that report, we had used SeqFeatR with the frequentist approach for the discovery. In Fig 3 we compare the latter approach (without correction for multiple testing) and Bayes factors with precision hyperparameters *K* = 1 and *K*_*D*_. For this comparison, we have chosen two significant associations identified in Ref. [24], namely the strongest (alignment position 66 with HLA type A*01, corresponding to position 38 of HBV core protein reference) and the weakest (alignment position 96 with HLA type B*44, corresponding to position 67 of HBV core protein reference).

For all three analyses of the association with HLA A*01, alignment position 66 clearly sticks out with extremely small *p* value and high values of *BF*_*K* = 1_, and *BF*_*K*_*D*__ (top row of Fig 3). A frequentist would not seriously consider any other position as associated with this HLA, and most of the positions have *p* ≈ 1. For *K* = 1 we have a wide spread around *BF* = 1, or log_10_ *BF* = 0. Two BFs other than at position 66 lie slightly above *BF* = 10 (or log_10_ *BF* = 1), a threshold often used to mark “substantial” evidence [25]. However, in contrast to the BF at position 66, these two BFs are not clearly separated from the bulk of the other BFs. Towards lower BFs, many values reach down to 10^−2^ or lower, indicating preference for independence over association at these alignment positions. For *K*_*D*_ we see the noise suppression mentioned earlier as the spread of the low BFs is constrained to a much smaller range than for *K* = 1.

For feature HLA B*44 we had only about 21 sequences (compared to 41 for HLA*A01), leading to a weaker association signal (bottom row of Fig 3). Still, the frequentist analysis shows position 96 with a *p* value that is clearly separated from the rest (panel D). However, a Bonferroni correction collapses all *p* values to 1, while the FDR correction collapses all to 1, except for position 96 with a corrected value of 0.16 (S1 Fig). The BFs with *K* = 1 do not favor association at any position (panel E). Conversely, for *K*_*D*_ position 96 has a clearly elevated BF (panel F). In summary, the frequentist approach with a strict correction for multiple comparisons, or the BF approach with *K* = 1 would both have led to a missing of the experimentally validated association at position 96, while the frequentist approach without correction, or *BF*_*K*_*D*__ both identify this association.

### Detection of phylogenetic bias

Sequences analyzed with SeqFeatR can often be considered samples from different branches of the same phylogenetic tree, evolved from a common ancestor under selection pressure related to the “feature”. A good example are again viral genome sequences evolved under selection pressure by the HLA systems (= features) of infected persons [8, 9, 24]. Under these circumstances, it is possible that SeqFeatR reports apparent sequence-feature associations that are due to a phylogenetic bias in the data. For instance, consider transmission of a virus from a mother to several children, all having the same HLA type. In this case, not only HLA escape mutations of viral proteins are associated with this HLA type, but apparently also mutations specific to the founder virus of the mother that are transmitted to the children, but unrelated to the HLA type. A mutation of a viral protein that is really associated with HLA type should co-occur with the HLA in other parts of the phylogenetic tree (i.e. outside this mother-child transmission), while this repeated co-occurrence is less likely for mutations that, due to phylogenetic bias, are only apparently associated with HLA.

**Fig 4:**
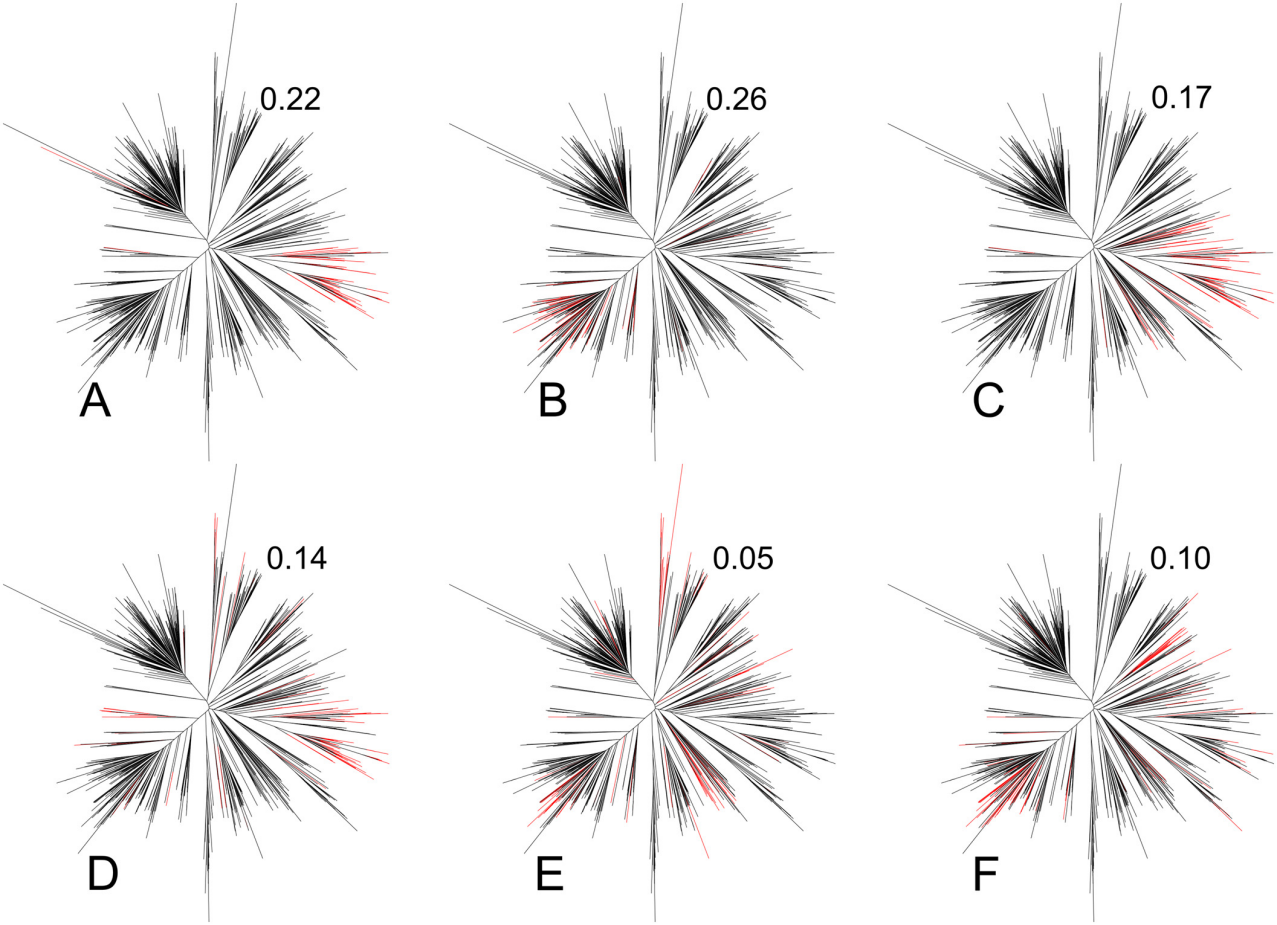
Phylogenetic distribution of feature-carrying sequences and phylogenetic bias indicator *B*. The distance-based phylogenetic tree in all six panels was computed for the same set of 788 East Asian HIV-1 gag protein sequences obtained from the HIV sequence database at http://www.hiv.lanl.gov. In each panel, those branches are colored red that correspond to sequences that carry an amino acid substitution apparently associated with a certain HLA type. The numbers to the upper right of each tree are the corresponding values of the bias indicator *B*, Eq (4).

SeqFeatR computes a simple quantitative indicator *B* of the strength of the phylogenetic bias for a given feature as follows. We expect that a phylogenetic bias is likely, if evolutionary distances within the group of sequences that carry the feature are much smaller than typical evolutionary distances in the total set of analyzed sequences. Thus, we define *B* as

$$\begin{array}{c} B=1-\frac{{\left\langle d_{ij}\right\rangle }_{feature}}{{\left\langle d_{ij}\right\rangle }_{all}}, \end{array}$$ (4)

where *d*_*ij*_ is the Levenshtein distance between sequences *i* and *j*. The ratio gives the mean distance between sequences carrying the feature over the mean distance in the total sequence sample. *B* lies then between values that typically are close to zero or even become negative for low bias, and a maximum of 1 for the strongest bias. For instance, in Fig 4E, feature-carrying sequences are spread out over different parts of the phylogenetic tree of all sequences in the sample, and consistent with this *B* = 0.05 signals low bias. Conversely, in Fig 4B feature-carrying leaves are concentrated in a sub-tree, and *B* = 0.26 indicates higher bias.

If detection of specific substitutions is desired that are associated with the feature, and not due to phylogenetic bias, a high *B* suggests extension of the set of sequences, especially with evolutionarily less closely related sequences that carry the feature.

### Examples beyond HLA-sequence association: HIV-1 co-receptor tropism and genetic species differences

In the above examples we have focused on the HLA type as feature and amino acid sequences. However, SeqFeatR is agnostic about the type of feature and sequence and therefore can be applied to other features and nucleotide sequences, too. To illustrate this we give in the following two examples.

#### HIV-1 co-receptor tropism

The Human Immunodeficiency Virus 1 (HIV-1) enters cells after contact with the cellular receptor CD4 and one of two co-receptors, either CCR5 or CXCR4 [26]. The choice of the co-receptor (or “co-receptor tropism”) is encoded in the viral genome, specifically in the third variable loop (V3) of the viral glycoprotein 120 [27]. Since the co-receptor tropism has implications for prognosis [28] and therapy [29], its determination from V3 sequence has attracted a lot of interest. Here we demonstrate that SeqFeatR recovers V3 sequence patterns known to be associated with co-receptor tropism.

To simplify the alignment, we used only V3 sequences of 35 amino acids (S1 Alignment), the by far most frequent length, from a dataset published earlier [30]. This led to 84 V3 sequences of CXCR4-tropic virus and 928 V3 sequences of CCR5-tropic virus. We then applied SeqFeatR with co-receptor tropism as feature. The resulting Manhattan plot (S2 Fig) shows many positions with highly significant deviations between CXCR4- and CCR5-tropic virus. One of the patterns recognized early on as specific for CXCR4 is the occurrence of positively charged amino acids at positions 11 and 25, the so-called 11/25 rule [31]. In fact, in the SeqFeatR output both positions 11 and 25 have significant deviations between CXCR4- and CCR5-tropic virus with p-values less than 10^−4^. Inspection of the alignment confirms that in CXCR4-tropic virus both positions 11 and 25 are significantly enriched in positively charged amino acids Arginine and Lysine in comparison to CCR5-tropic virus.

#### Genetic species differences

SeqFeatR can be used to discover genetic differences between species or other taxonomic levels. For the following example we have retrieved from the SILVA database [32], version 123, RNA sequences of the small ribosomal subunit (SSU) of two closely related green algae, *Chlamydomonas applanata* (9 sequences) and *Chlamydomonas reinhardtii* (10 sequences). The input alignment is provided as S2 Alignment. Using these two species as features, we found with SeqFeatR 29 positions with highly significant differences (red stars in Manhattan plot S3 Fig). Nucleotide sequence differences such as these can be used to understand genetic bases of species differences or to design species specific PCR primers [33].

### SeqFeatR addresses various needs and levels of expertise

SeqFeatR has three modes of use, addressing users with different levels of expertise and different needs: For users not versed in R programming and with sequence material and features that can be transmitted over the Internet, we offer the SeqFeatR web server. For reproducibility and documentation, the web server generates a detailed report for the user. If the data must not leave the respective institution, inexperienced users may still use a simple Tcl/Tk-based graphical user interface (GUI) that can be started by the SeqFeatR_GUI() command from R. Experienced users can access the full range of SeqFeatR commands in R-scripts. Training material such as tutorial texts (https://cran.r-project.org/web/packages/SeqFeatR/vignettes/SeqFeatR_tutorial.pdf) and videos are provided for users at all levels.

## Supporting Information

S1 Fig Frequentist approach with correction for multiple testing. Association of alignment positions of HBV core protein with patient HLA types A*01 (**A**) and B*44 (**B**). Sequence numbers in panel titles are feature-carrying fractions of the total of 148 sequences included in the alignment. Association of sequences with feature HLA were analyzed with Fisher’s exact test, and resulting *p* values were corrected for multiple testing with FDR option.
(TIFF)

S2 Fig Association of V3 sequence positions with HIV-1 co-receptor tropism. Manhattan plot output of SeqFeatR showing sites in the V3 amino acid sequences S1 Alignment that are significantly associated with co-receptor tropism.
(PDF)

S3 Fig Association of *Chlamydomonas* SSU nucleotide sequence position with species. Manhattan plot output of SeqFeatR showing sites in the SSU nucleotide sequence alignment S2 Alignment that are significantly associated with *Chlamydomonas* species, here: *Chlamydomonas reinhardtii* (RH) vs *Chlamydomonas applanata* (AP).
(PDF)

S1 Alignment V3 amino acid sequences of CCR5- and CXCR4-tropic HIV-1. S2 Fig was produced by SeqFeatR with this input. All sequences (84 from CXCR4-tropic and from 928 CCR5-tropic virus) have the same length of 35 amino acids and have not been submitted to an extra alignment step. Note that the feature labels “X4” (for CXCR4-tropic) and “R5” (for CCR5-tropic) have been added at the end of the FASTA headers after a semicolon.
(FA) Click here for additional data file.

S2 Alignment Alignment of SSU nucleotide sequences from *Chlamydomonas*. Alignment of RNA sequences of small ribosomal subunit sequences: 9 from *Chlamydomonas applanata*, 10 from *Chlamydomonas reinhardtii*. S3 Fig was generated by SeqFeatR with this input. Note again that the last element of the FASTA header stands for the feature, here: RH for *reinhardtii* and AP for *applanata*.
(FA) Click here for additional data file.
