# Supplementary figures and images for: SeqFeatR for the Discovery of Feature-Sequence Associations
Source: PLoS One. 2016 Jan 5;11(1):e0146409. doi: 10.1371/journal.pone.0146409 (PMC4701496; doi:10.1371/journal.pone.0146409)

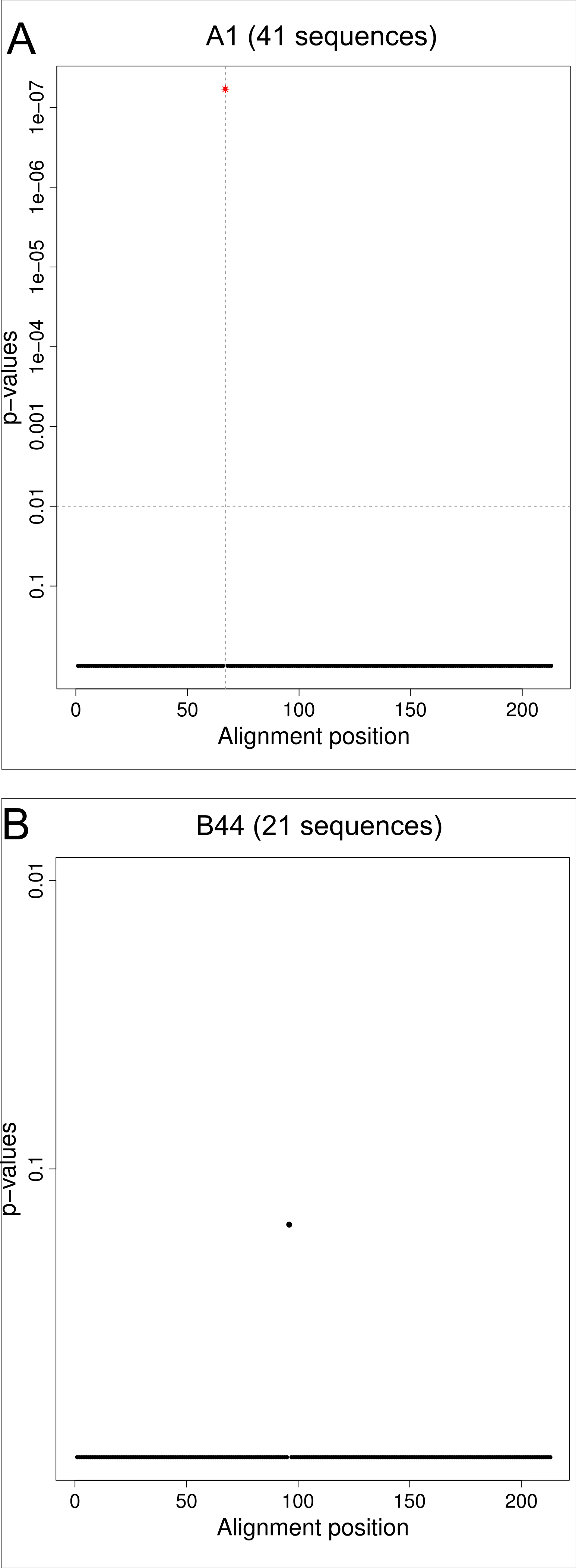

Supplement: S1 Fig — Association of alignment positions of HBV core protein with patient HLA types A*01 (A) and B*44 (B). Sequence numbers in panel titles are feature-carrying fractions of the total of 148 sequences included in the alignment. Association of sequences with feature HLA were analyzed with Fisher’s exact test, and resulting p values were corrected for multiple testing with FDR option. (TIFF) [file pone.0146409.s001.tiff]

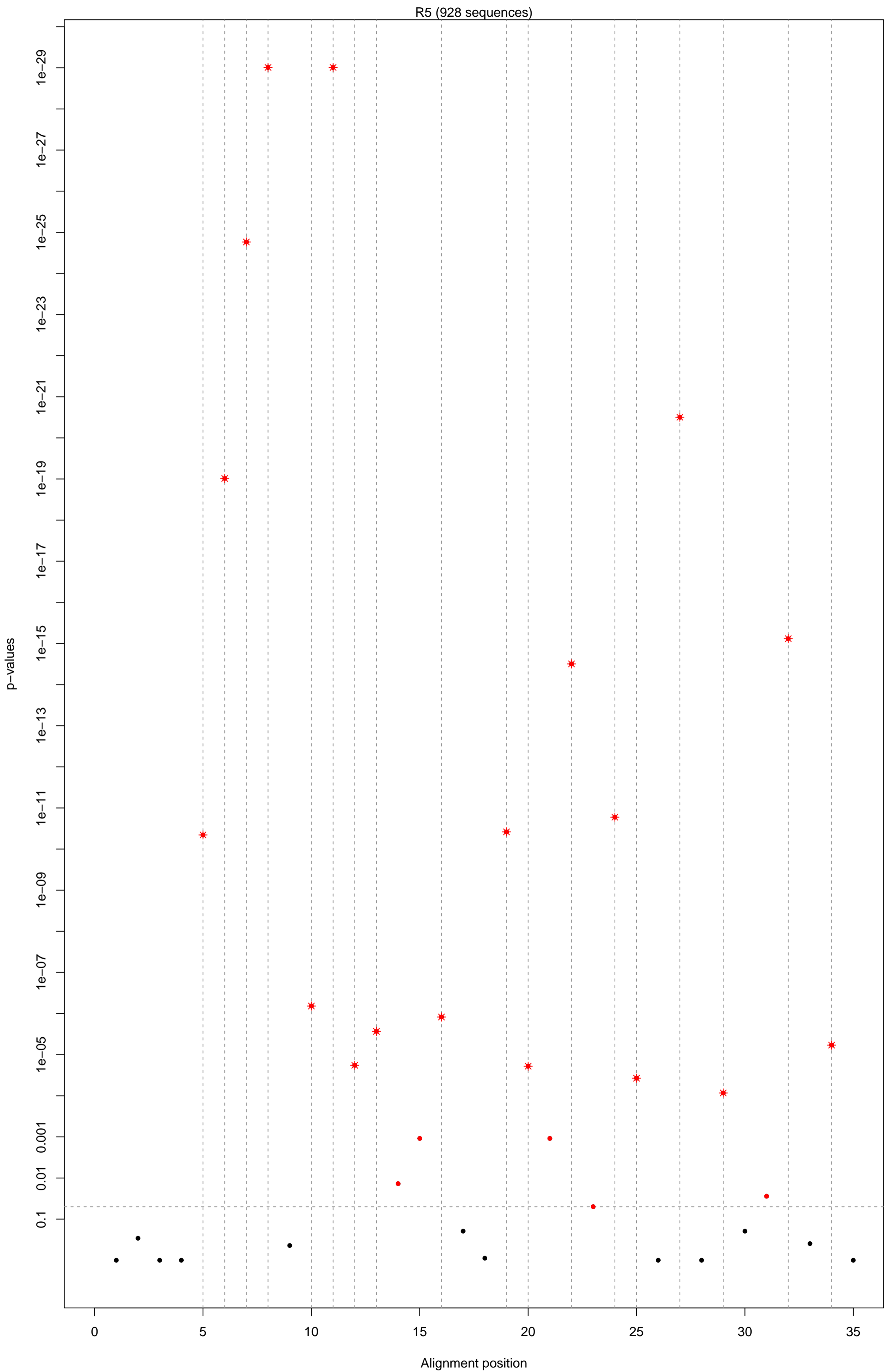

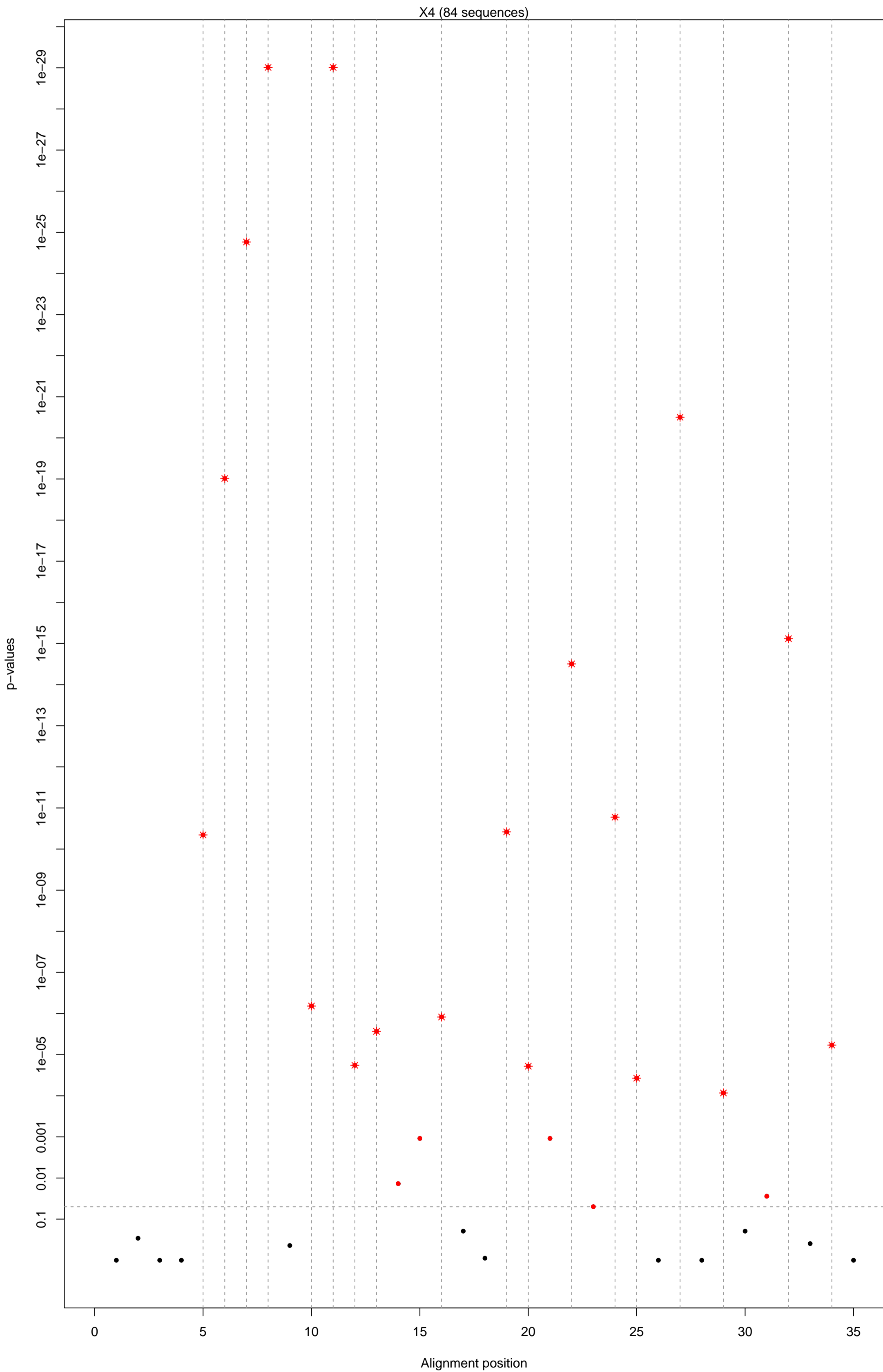

Supplement: S2 Fig — Manhattan plot output of SeqFeatR showing sites in the V3 amino acid sequences S1 Alignment that are significantly associated with co-receptor tropism. (PDF) [file pone.0146409.s002.pdf]

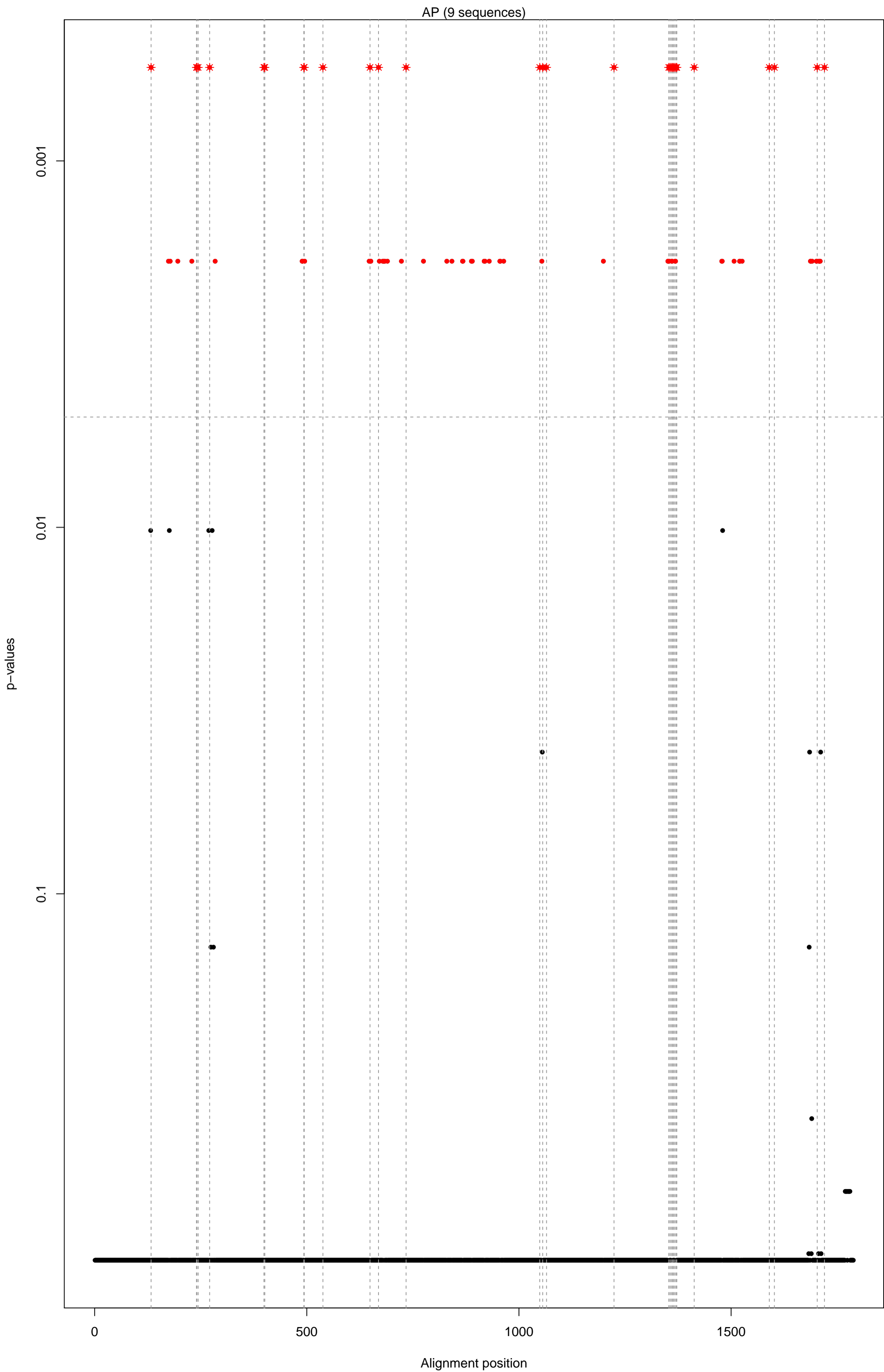

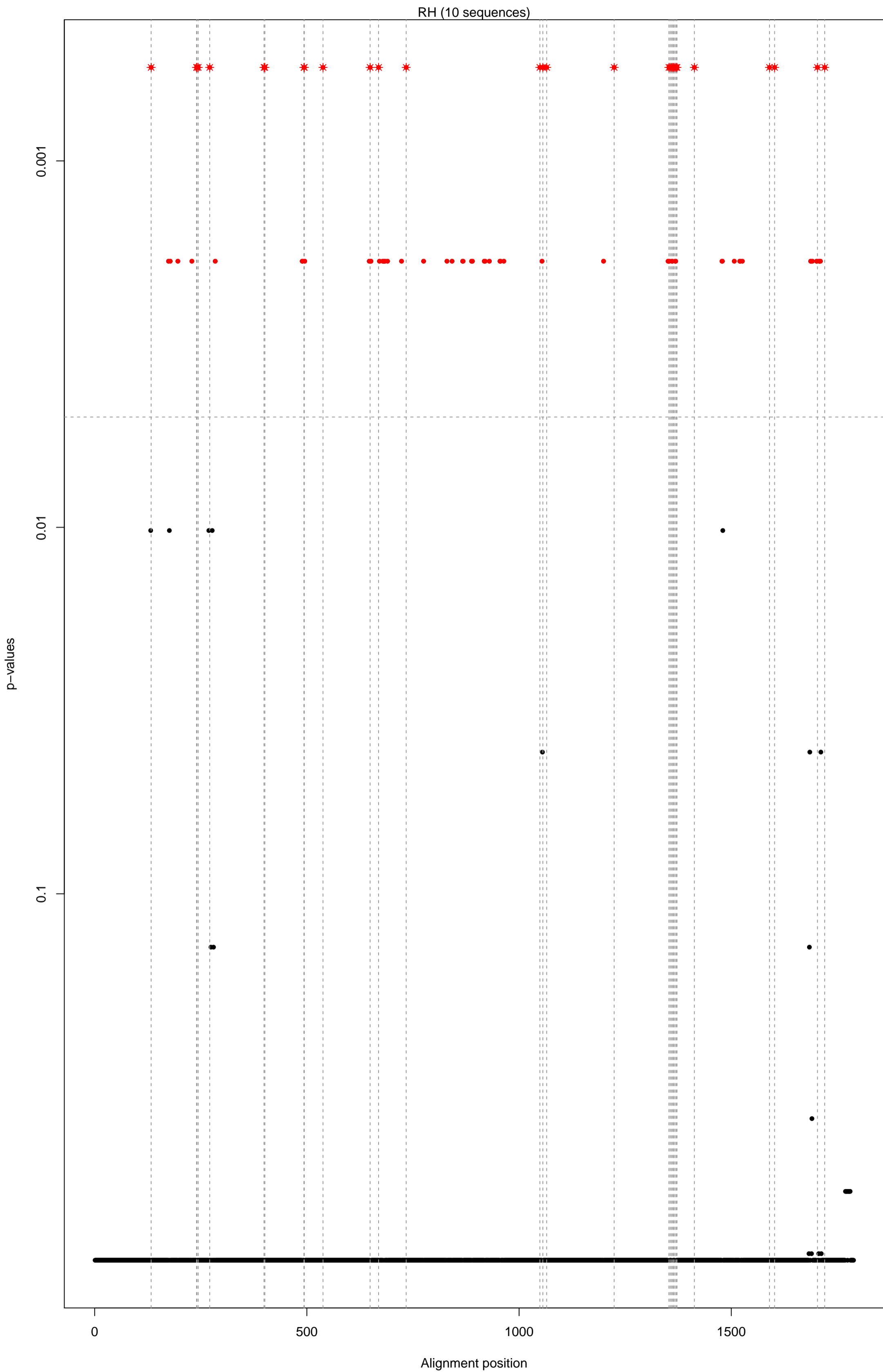

Supplement: S3 Fig — Manhattan plot output of SeqFeatR showing sites in the SSU nucleotide sequence alignment S2 Alignment that are significantly associated with Chlamydomonas species, here: Chlamydomonas reinhardtii (RH) vs Chlamydomonas applanata (AP). (PDF) [file pone.0146409.s003.pdf]
